# Correction: Stapled histone H3 tails are super-substrates for lysine methyltransferase SETD7

**DOI:** 10.1039/d6sc90024k

**Published:** 2026-02-03

**Authors:** Nurgül Bilgin, Laust Moesgaard, Jacob Kongsted, Jasmin Mecinović

**Affiliations:** a Department of Physics, Chemistry and Pharmacy, University of Southern Denmark Campusvej 55 5230 Odense Denmark mecinovic@sdu.dk

## Abstract

Correction for ‘Stapled histone H3 tails are super-substrates for lysine methyltransferase SETD7’ by Nurgül Bilgin *et al.*, *Chem. Sci.*, 2026, https://doi.org/10.1039/d5sc08094k.

Upon publication of the original article, the authors were made aware of an additional previous work that warranted inclusion in the prior cited literature.

In a prior work by Jeltsch *et al.*^1^ it was reported that the introduction of cysteine residues at the same −3 and +2 positions of SETD7 target peptides increased the activity of SETD7. The effect was observed only after introducing both cysteine residues, indicating that a disulfide bond is generated. The finding was correlated with the crystal structure of SETD7 containing the H3 substrate peptide in a hairpin conformation and concluded that the higher enzymatic activity was due to a stabilization of the substrate peptide in the loop conformation through disulfide bond formation. In the same work, the authors demonstrated dimethylation of a peptide substrate derived from the MINT protein by SETD7, while the H3K4 peptide was only monomethylated.

The Royal Society of Chemistry apologises for these errors and any consequent inconvenience to authors and readers.
